# Disparities in Hemoglobin A_1c_ Levels in the First Year After Diagnosis Among Youths With Type 1 Diabetes Offered Continuous Glucose Monitoring

**DOI:** 10.1001/jamanetworkopen.2023.8881

**Published:** 2023-04-19

**Authors:** Ananta Addala, Victoria Ding, Dessi P. Zaharieva, Franziska K. Bishop, Alyce S. Adams, Abby C. King, Ramesh Johari, David Scheinker, Korey K. Hood, Manisha Desai, David M. Maahs, Priya Prahalad

**Affiliations:** 1 Division of Pediatric Endocrinology, Department of Pediatrics, Stanford University, Stanford, California; 2 Division of Biomedical Informatics Research, Department of Medicine, Stanford University, Stanford, California; 3 Department of Epidemiology and Population Health, Stanford University School of Medicine, Stanford, California; 4 Department of Health Policy, Stanford University School of Medicine, Stanford, California; 5 Stanford Diabetes Research Center, Stanford University, Stanford, California; 6 Stanford Prevention Research Center Division, Department of Medicine, Stanford University School of Medicine, Stanford, California; 7 Clinical Excellence Research Center, Stanford University, Stanford, California; 8 Department of Management Science and Engineering, Stanford University, Stanford, California

## Abstract

**Importance:**

Continuous glucose monitoring (CGM) is associated with improvements in hemoglobin A_1c_ (HbA_1c_) in youths with type 1 diabetes (T1D); however, youths from minoritized racial and ethnic groups and those with public insurance face greater barriers to CGM access. Early initiation of and access to CGM may reduce disparities in CGM uptake and improve diabetes outcomes.

**Objective:**

To determine whether HbA_1c_ decreases differed by ethnicity and insurance status among a cohort of youths newly diagnosed with T1D and provided CGM.

**Design, Setting, and Participants:**

This cohort study used data from the Teamwork, Targets, Technology, and Tight Control (4T) study, a clinical research program that aims to initiate CGM within 1 month of T1D diagnosis. All youths with new-onset T1D diagnosed between July 25, 2018, and June 15, 2020, at Stanford Children’s Hospital, a single-site, freestanding children’s hospital in California, were approached to enroll in the Pilot-4T study and were followed for 12 months. Data analysis was performed and completed on June 3, 2022.

**Exposures:**

All eligible participants were offered CGM within 1 month of diabetes diagnosis.

**Main Outcomes and Measures:**

To assess HbA_1c_ change over the study period, analyses were stratified by ethnicity (Hispanic vs non-Hispanic) or insurance status (public vs private) to compare the Pilot-4T cohort with a historical cohort of 272 youths diagnosed with T1D between June 1, 2014, and December 28, 2016.

**Results:**

The Pilot-4T cohort comprised 135 youths, with a median age of 9.7 years (IQR, 6.8-12.7 years) at diagnosis. There were 71 boys (52.6%) and 64 girls (47.4%). Based on self-report, participants’ race was categorized as Asian or Pacific Islander (19 [14.1%]), White (62 [45.9%]), or other race (39 [28.9%]); race was missing or not reported for 15 participants (11.1%). Participants also self-reported their ethnicity as Hispanic (29 [21.5%]) or non-Hispanic (92 [68.1%]). A total of 104 participants (77.0%) had private insurance and 31 (23.0%) had public insurance. Compared with the historical cohort, similar reductions in HbA_1c_ at 6, 9, and 12 months postdiagnosis were observed for Hispanic individuals (estimated difference, −0.26% [95% CI, −1.05% to 0.43%], −0.60% [−1.46% to 0.21%], and −0.15% [−1.48% to 0.80%]) and non-Hispanic individuals (estimated difference, −0.27% [95% CI, −0.62% to 0.10%], −0.50% [−0.81% to −0.11%], and −0.47% [−0.91% to 0.06%]) in the Pilot-4T cohort. Similar reductions in HbA_1c_ at 6, 9, and 12 months postdiagnosis were also observed for publicly insured individuals (estimated difference, −0.52% [95% CI, −1.22% to 0.15%], −0.38% [−1.26% to 0.33%], and −0.57% [−2.08% to 0.74%]) and privately insured individuals (estimated difference, −0.34% [95% CI, −0.67% to 0.03%], −0.57% [−0.85% to −0.26%], and −0.43% [−0.85% to 0.01%]) in the Pilot-4T cohort. Hispanic youths in the Pilot-4T cohort had higher HbA_1c_ at 6, 9, and 12 months postdiagnosis than non-Hispanic youths (estimated difference, 0.28% [95% CI, −0.46% to 0.86%], 0.63% [0.02% to 1.20%], and 1.39% [0.37% to 1.96%]), as did publicly insured youths compared with privately insured youths (estimated difference, 0.39% [95% CI, −0.23% to 0.99%], 0.95% [0.28% to 1.45%], and 1.16% [−0.09% to 2.13%]).

**Conclusions and Relevance:**

The findings of this cohort study suggest that CGM initiation soon after diagnosis is associated with similar improvements in HbA_1c_ for Hispanic and non-Hispanic youths as well as for publicly and privately insured youths. These results further suggest that equitable access to CGM soon after T1D diagnosis may be a first step to improve HbA_1c_ for all youths but is unlikely to eliminate disparities entirely.

**Trial Registration:**

ClinicalTrials.gov Identifier: NCT04336969

## Key Points

**Question:**

Is inclusive initiation of continuous glucose monitoring (CGM) at diagnosis associated with reduced disparities in glycemic control among youths with type 1 diabetes?

**Findings:**

In this cohort study of 135 youths with new-onset type 1 diabetes, similar improvements in hemoglobin A_1c_ following CGM initiation were observed irrespective of ethnicity or insurance status. However, hemoglobin A_1c_ levels remained higher among Hispanic participants and youths with public insurance compared with their counterparts.

**Meaning:**

These results suggest that expanding access to CGM is a potential strategy for improving glycemic outcomes and reducing disparities but requires broader societal strategies to address structural drivers of disparities in diabetes care.

## Teaser

This cohort study assesses disparities in hemoglobin A_1c_ (HbA_1c_) levels by ethnicity and insurance status among youths with new-onset type 1 diabetes offered continuous glucose monitoring (CGM).

## Introduction

Continuous glucose monitoring (CGM) is an effective tool to improve glycemic outcomes and quality of life for youths with type 1 diabetes (T1D).^1,2^ Rates of diabetes technology use are 50% lower among youths with T1D from lower-income backgrounds despite higher rates of complications in this group.^3,4,5,6,7^ In recent decades, the hemoglobin A1c (HbA_1c_) gap has worsened and is partially explained by differences in technology uptake among youths from historically marginalized groups.^3,4^ Restrictions on CGM coverage by public insurance are a critical barrier to access among low-income youths and youths from minoritized racial and ethnic groups.^3,5,6,8^ A substantial portion of youths with public insurance have frequent interruptions to their CGM access due to insurance- and payer-related issues and these interruptions are associated with worsening HbA_1c_ trends.^5^ Diabetes clinicians’ implicit biases and willingness to recommend diabetes technology to youths from underrepresented backgrounds are an additional likely contributor to disparities.^9,10,11,12,13^ Additionally, social determinants of health are hypothesized to be key drivers in technology access and utilization.^14,15^ Equitable access to and use of diabetes technology is a potential strategy to reduce disparities in diabetes care by socioeconomic status and race and ethnicity.^3,4,16^

The Teamwork, Targets, Technology, and Tight Control (4T) study is a pragmatic clinical research program at Stanford Children’s Hospital to initiate CGM within 1 month of diagnosis. The study approached all patients with new-onset T1D and guaranteed access to CGM supplies for the study duration, thereby addressing clinician- and insurance-mediated barriers.^3,5,9,13,17,18,19^ In this exploratory analysis, we compared HbA_1c_ trajectories, stratified by ethnicity and insurance, for youths in the Pilot-4T cohort compared with a historical cohort within our clinic. We hypothesized that by implementing a study protocol intending to address clinician- and insurance-mediated barriers, Hispanic youths and youths with public insurance would achieve (1) substantial improvements in HbA_1c_ compared with their historical counterparts and (2) HbA_1c_ values that remained higher than their non-Hispanic and privately insured peers due to the influence of other social determinants of health.

## Methods

### Study Design

The Stanford University Institutional Review Board approved the protocols and procedures used in this cohort study. Institutionally approved informed consent was obtained for all participants. If the study participant was aged younger than 18 years, assent was obtained from youths and consent was obtained from their parent or guardian. The study followed the Strengthening the Reporting of Observational Studies in Epidemiology (STROBE) reporting guideline.

The Pilot-4T study protocol has been described previously.^20,21,22,23^ Briefly, all youths with newly diagnosed T1D were approached to enroll in the Pilot-4T study to initiate CGM with the Dexcom G6 system (Dexcom) within 1 month of diagnosis. The Pilot-4T cohort comprised youths newly diagnosed with T1D between July 25, 2018, and June 15, 2020. Starting in March 2019, 89 participants were additionally offered weekly remote monitoring of CGM data by certified diabetes care and education specialists, with insulin dose changes sent via a secure patient-clinician messaging platform supported by the MyChart electronic medical system (Epic). Certified diabetes care and education specialists supported patients with diabetes management, provided CGM-specific education and troubleshooting assistance, and answered concerns via MyChart as needed.

### Participants and Cohort Descriptions

The Pilot-4T cohort was followed from diagnosis date (baseline) to 1 of 3 end points: CGM discontinuation date, study withdrawal, or study end (June 30, 2021). These participants were compared with a historical cohort^24^ that included youths diagnosed with T1D at Stanford Children’s Hospital between June 1, 2014, and December 28, 2016. The historical cohort received the clinical standard of care at that time, which included new-onset diabetes education, quarterly clinic visits, and a nonstandardized introduction to CGM at the discretion of the clinician and family.

### Ethnicity and Insurance Variables

Participant race and ethnicity data were gathered via self-report following principles for collecting and reporting race and ethnicity in research.^25^ For participants with missing race and ethnicity variables, study staff prompted participants to complete the self-report survey and supplemented it with data abstraction from the electronic medical record. Any participants with missing race and ethnicity data were excluded from the analyses.

Race and ethnicity variables were structured consistent with the US Census, with self-reported ethnicity (Hispanic or non-Hispanic) and race (American Indian or Alaska Native, Asian, Black, Native Hawaiian or other Pacific Islander, White, or other race [or multiple races if >1 selected]) presented separately.^25,26^ Participants self-reported their ethnicity identification as Hispanic or non-Hispanic. In our clinic, 25.6% of patients identified as Hispanic or Latino and 60.6% identified as non-Hispanic, with 13.8% missing an ethnicity designation. In California, non-Hispanic individuals represented in our cohort (Asian, non-Hispanic White, and multiple races) have similar socioeconomic status.^26,27^ Given the similar sociodemographic characteristics in California and Stanford Children’s Hospital, we opted to analyze our cohort by ethnicity (non-Hispanic vs Hispanic) to evaluate differences among youths from minoritized ethnic groups.

Participant insurance type (public vs private) was determined by review of the electronic medical record. Public insurance was identified when the primary insurance was Medi-Cal/Medicaid, Medicare, or California Children’s Services.

### Study Outcomes

The primary outcome was change in HbA_1c_ from 4 months (established nadir of HbA_1c_ for the historical cohort^22^) to 12 months postdiagnosis. Secondary outcomes included the proportion of participants achieving the target HbA_1c_ levels of less than 7.5% and less than 7.0% according to American Diabetes Association guidelines at study initiation^28^ and data analysis,^29^ respectively. Exploratory outcomes consisted of CGM metrics, including sensor glucose time in range (TIR; 70-180 mg/dL), hypoglycemia (54-69 mg/dL), and clinically significant hypoglycemia (<54 mg/dL) (to convert glucose to mmol/L, multiply by 0.0555). The CGM wear-time was calculated from glucose data points available from the CGM start date to 1 year after diagnosis available in the Dexcom Clarity portal. A limitation to this method is the potential difference in available data points when using Dexcom receivers vs smart devices due to the cloud and internet connectivity of download devices.^30^ Therefore, we conducted a secondary analysis evaluating use-time by download device stratified by ethnicity and insurance.

Assessment of HbA_1c_ was performed using a DCA Vantage Analyzer (Siemens Medical Solutions USA). Due to the increase in virtual telehealth visits during the COVID-19 pandemic, we incorporated home HbA_1c_ measurements in November 2020 (analyzed by the University of Minnesota Advanced Research and Diagnostic Laboratory).^31,32^

### Statistical Analysis

Baseline and follow-up characteristics were summarized by ethnicity (Hispanic vs non-Hispanic) and insurance status (public vs private). Group differences in baseline characteristics were evaluated by standardized mean differences (SMDs) to assess small (<0.2), medium (0.5), and large (0.8) effect sizes.^33^ All who initiated CGM in the first year were included in this analysis under the intention-to-treat principle.

Differences in HbA_1c_ trajectories of the Pilot-4T cohort were visualized by ethnicity and insurance using locally estimated scatterplot smoothing (LOESS), with similarly stratified historical trajectories as the benchmark. Differences in LOESS means between the Pilot-4T cohort and the historical cohort were calculated at 6, 9, and 12 months. Differences in LOESS means at each time point are presented with bootstrapped 95% CIs from 1000 resamples on the participant level. The level of smoothing in LOESS was determined by the span parameter, where we selected the value that minimized the mean squared error via 10-fold cross-validation. The proportions of the cohort with HbA_1c_ levels of less than 7.5% and less than 7.0%, respectively, are presented descriptively using bar plots over time. Exploratory outcomes of CGM metrics (mean CGM glucose, hypoglycemia, and TIR) for the first 12 months after diagnosis were visualized using LOESS and stacked bar plots over time. The CGM data were systematically collected for youths in the 4T cohort but not for the historical cohort, which had a limited and nonsystematic approach to CGM use. Thus, CGM metrics were only analyzed for the 4T cohort.

For the comparisons of each primary key variable of interest (ethnicity and insurance), a linear mixed-effects regression model that allowed for 2 piecewise linear slopes of HbA_1c_ levels to be estimated from diagnosis to 4 months postdiagnosis (nadir in HbA_1c_) and from 4 to 12 months postdiagnosis was used to calculate cohort differences in 4- to 12-month slopes assessed via an interaction term, the main parameter of interest. Within-patient correlation of HbA_1c_ was accounted for using a patient-specific random effect; both models were adjusted for age and sex, with ethnicity and insurance comparisons additionally adjusted for these 2 variables, respectively. A 2-sided Wald test was used to test the interaction term, with significance assessed at an α level of .05. For all analyses, statistical significance was assessed at an α level of .05 (2-tailed).

All analyses were conducted using R, version 4.0 (R Project for Statistical Computing).^34^ Data analysis was performed and completed on June 3, 2022.

## Results

### Cohort Characteristics

The Pilot-4T cohort study enrolled 135 of 146 eligible youths (8 declined, 2 transferred care, and 1 did not meet inclusion criteria). Their median age at diagnosis was 9.7 years (IQR, 6.8-12.7 years). There were 71 boys (52.6%) and 64 girls (47.4%); 104 (77.0%) had private insurance and 31 (23.0%) had public insurance. Based on self-report, participants’ race was categorized as Asian or Pacific Islander (19 [14.1%]), White (62 [45.9%]), or other race (39 [28.9%]); race was missing or not reported for 15 participants (11.1%). Participants also self-reported their ethnicity as Hispanic (29 [21.5%]) or non-Hispanic (92 [68.1%]). Pilot-4T participants had a mean (SD) HbA_1c_ of 12.2% (2.1%) at diagnosis, with a median CGM initiation of 7 days (range, 5-11 days).

A historical cohort of 272 youths was compared with the Pilot-4T cohort. The historical cohort had a median age of 9.7 years (range, 6.7-12.7 years) at diagnosis. There were 137 boys (50.4%) and 135 girls (49.6%); 197 (72.4%) had private insurance. Historical cohort participants self-reported their race and ethnicity as American Indian or Alaska Native (1 [0.4%]), non-Hispanic Black (5 [1.8%]), or non-Hispanic White (116 [42.6%]). The historical cohort had a mean (SD) HbA_1c_ of 10.7% (2.5%) at diagnosis, with 56.2% CGM use (<1.8% started CGM ≤30 days of diagnosis^22^).

Baseline and follow-up characteristics of the Pilot-4T cohort are provided in the Table. Group differences in baseline characteristics by ethnicity and insurance status were observed (eTable in Supplement 1). Large SMDs were observed for Hispanic vs non-Hispanic youths stratified by public insurance (17 [58.6%] vs 12 [13.0%]; SMD, 1.08 [95% CI, 0.64-1.5]) and English as the primary language (17 [58.6%] vs 86 [93.5%]; SMD, 0.90 [95% CI, 0.46-1.3]). Moderate SMDs were observed by public vs private insurance for median age (11.0 years [IQR, 8.6-14.6 years] vs 9.3 years [IQR, 5.9-12.1 years]; SMD, 0.45 [95% CI, 0.05-0.86]) and English as the primary language (22 [71.0%] vs 95 [91.3%]; SMD, 0.54 [95% CI, 0.13-0.95]). Group differences in additional characteristics were also observed, including median days to CGM initiation by public vs private insurance status (10 days [IQR, 7-13 days] vs 7 days [IQR, 5-11 days]). The CGM wear-time also varied by Hispanic vs non-Hispanic ethnicity (52.2% [IQR, 16.1%-73.1%] vs 94.0% [IQR, 80.9%-98.0%]) and by public vs private insurance (62.1% [IQR, 18.8%-80.8%] vs 93.9% [IQR, 82.4%-98.0%]). On further evaluation, we found that use-time was lowest for individuals using the CGM receiver device compared with smart devices, irrespective of ethnicity or insurance (eFigure 1 in Supplement 1).

**Table.** Pilot-4T Cohort Demographics Stratified by Ethnicity and Insurance Status^a^

Characteristic	Overall (N = 135)	Ethnicity^b^			Insurance	
		Hispanic (n = 29)	Non-Hispanic (n = 92)	Unknown (n = 14)	Public (n = 31)	Private (n = 104)
Age at onset, y, median (IQR)	9.7 (6.8-12.7)	9.7 (9.0-13.9)	9.2 (5.7-11.9)	12.4 (8.5-14.2)	11.0 (8.6-14.6)	9.3 (5.9-12.1)
Sex						
Female	64 (47.4)	20 (69.0)	40 (43.5)	4 (28.6)	19 (61.3)	45 (43.3)
Male	71 (52.6)	9 (31.0)	52 (56.5)	10 (71.4)	12 (38.7)	59 (56.7)
Race^b^						
Asian or Pacific Islander	19 (14.1)	0	19 (20.7)	0	2 (6.5)	17 (16.3)
White	62 (45.9)	8 (27.6)	54 (58.7)	0	12 (38.7)	50 (48.1)
Other	39 (28.9)	20 (69.0)	18 (19.6)	1 (7.1)	13 (41.9)	26 (25.0)
Unknown or declined to state	15 (11.1)	1 (3.4)	1 (1.1)	13 (92.9)	4 (12.9)	11 (10.6)
HbA_1c_ at onset, %, mean (SD)	12.2 (2.1)	11.9 (1.9)	12.3 (2.2)	12.3 (1.9)	12.2 (2.4)	12.3 (2.0)
Insurance status						
Private	104 (77.0)	12 (41.4)	80 (87.0)	12 (85.7)	0	104 (100)
Public	31 (23.0)	17 (58.6)	12 (13.0)	2 (14.3)	31 (100)	0
Primary language						
English	117 (86.7)	17 (58.6)	86 (93.5)	14 (100)	22 (71.0)	95 (91.3)
Other	18 (13.3)	12 (41.4)	6 (6.5)	0	9 (29.0)	9 (8.7)
CGM initiation	135 (100)	29 (100)	92 (100)	14 (100)	31 (100)	104 (100)
Early (≤30 d)	124 (91.9)	25 (86.2)	88 (95.7)	11 (78.6)	26 (83.9)	98 (94.2)
Late (>30 d)	11 (8.1)	4 (13.8)	4 (4.3)	3 (21.4)	5 (16.1)	6 (5.8)
Days to CGM initiation, median (IQR)	7 (5-11)	9 (6-12)	7 (5-11)	11 (7-14)	10 (7-13)	7 (5-11)
CGM wear-time, %, median (IQR)^c^	92.2 (65.2-97.7)	52.2 (16.1-73.1)	94.0 (80.9-98.0)	93.4 (87.7-97.9)	62.1 (18.8-80.8)	93.9 (82.4-98.0)
Insulin pump use	72 (53.3)	10 (34.5)	53 (57.6)	9 (64.3)	14 (45.2)	58 (55.8)
Open loop	38 (28.1)	3 (10.3)	30 (32.6)	5 (35.7)	4 (12.9)	34 (32.7)
Predictive low glucose suspend	18 (13.3)	3 (10.3)	12 (13.0)	3 (21.4)	3 (9.7)	15 (14.4)
Advanced hybrid closed loop	32 (23.7)	6 (20.7)	21 (22.8)	5 (35.7)	9 (29.0)	23 (22.1)
None	63 (46.7)	19 (65.5)	39 (42.4)	5 (35.7)	17 (54.8)	46 (44.2)
Days to pump initiation, median (IQR)	212 (127-384)	285 (123-359)	201 (127-395)	195 (184-266)	274 (150-448)	186 (127-379)

Abbreviation: CGM, continuous glucose monitoring.

^a^ Unless indicated otherwise, data are presented as the No. (%) of youths.

^b^ Participants self-reported race as American Indian or Alaska Native, Asian, Black, Native Hawaiian or other Pacific Islander, White, or other race (or multiple races if >1 selected); for purposes of the analysis, race was categorized as Asian or Pacific Islander, White, or other.

^c^ Percentage of time CGM was worn out of eligible hours of device wear.

### HbA_1c_ Trajectories by Ethnicity and Insurance

**Figure 1.**
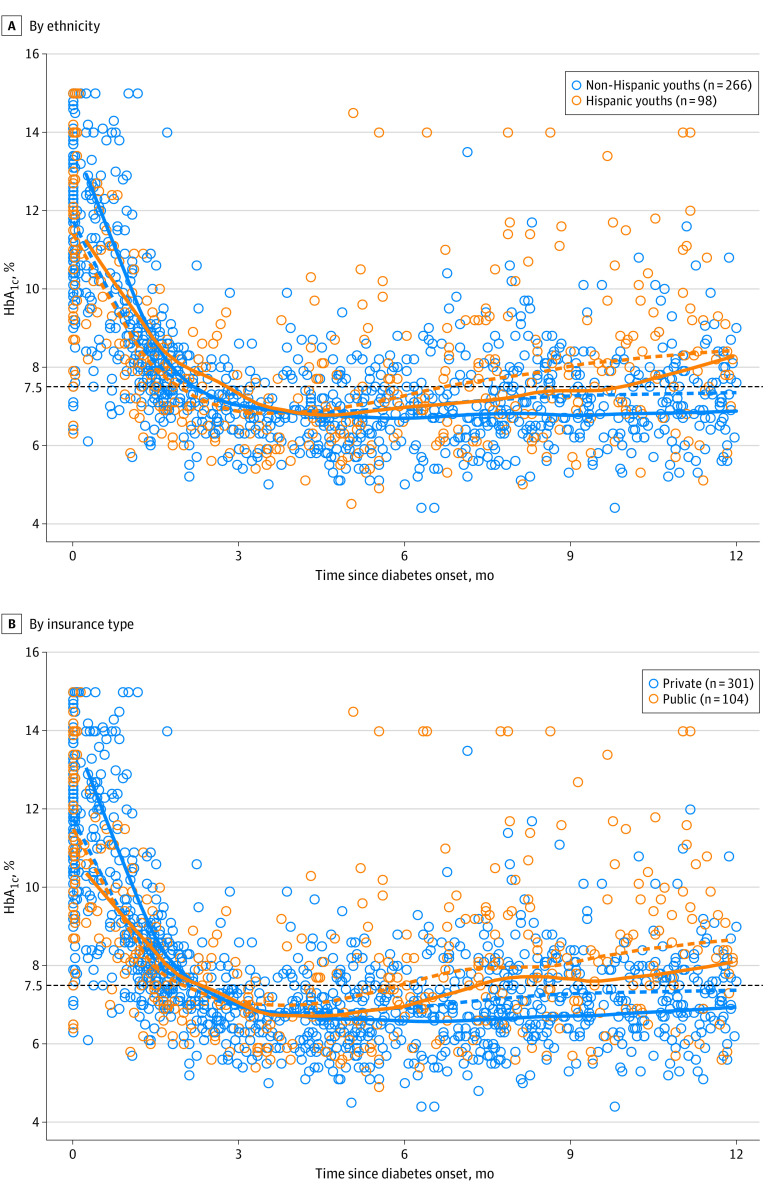
Hemoglobin A_1c_ (HbA_1c_) Values for Youths in the Pilot-4T and Historical Cohorts Over the First 12 Months by Ethnicity and Insurance Status A, Hispanic vs non-Hispanic ethnicity. B, Public vs private insurance. Solid lines represent the Pilot-4T cohort; dashed lines represent the historical cohort. The horizontal black dashed line indicates target HbA_1c_ level.

Compared with their historical counterparts, youths in the Pilot-4T cohort had improvements in HbA_1c_ irrespective of ethnicity and insurance (Figure 1). The HbA_1c_ nadir was observed at 4 months postdiagnosis with a subsequent increase from 4 to 12 months for all groups. At 6, 9, and 12 months postdiagnosis, HbA_1c_ was lower in the Pilot-4T cohort for Hispanic youths (estimated difference, −0.26% [95% CI, −1.05% to 0.43%], −0.60% [−1.46% to 0.21%], and −0.15% [−1.48% to 0.80%]) and non-Hispanic youths (estimated difference, −0.27% [95% CI, −0.62% to 0.10%], −0.50% [−0.81% to −0.11%], and −0.47% [−0.91% to 0.06%]) compared with the historical cohort. Similar trends were observed at 6, 9, and 12 months for youths with public insurance (estimated difference, −0.52% [95% CI, −1.22% to 0.15%], −0.38% [−1.26% to 0.33%], and −0.57% [−2.08% to 0.74%]) and for youths with private insurance (estimated difference, −0.34% [95% CI, −0.67% to 0.03%], −0.57% [−0.85% to −0.26%], and −0.43% [−0.85% to 0.01%]). Within the Pilot-4T cohort, lower HbA_1c_ at diagnosis but higher HbA_1c_ at 6, 9, and 12 months postdiagnosis was observed for Hispanic youths (estimated difference, 0.28% [95% CI, −0.46% to 0.86%], 0.63% [0.02% to 1.20%], and 1.39% [0.37% to 1.96%]; Figure 1A) and for youths with public insurance (estimated difference, 0.39% [95% CI, −0.23% to 0.99%], 0.95% [0.28% to 1.45%], and 1.16% [−0.09% to 2.13%]; Figure 1B) compared with their counterparts.

**Figure 2.**
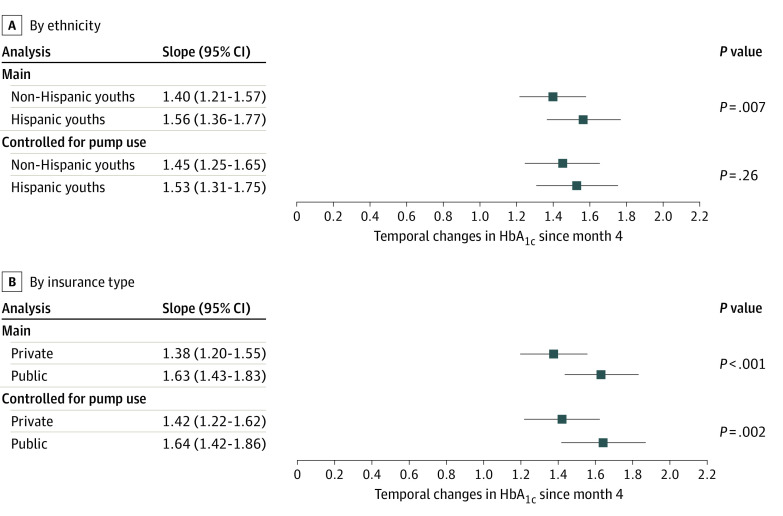
Linear Mixed-Effects Regression Model of Linear Slopes Metrics for Youths in the Pilot-4T Cohort Comparing Hemoglobin A_1c_ (HbA_1c_) Increase by Ethnicity and Insurance Status A, Hispanic vs non-Hispanic ethnicity. B, Public vs private insurance. The analysis is adjusted for age and sex, with ethnicity and insurance status comparisons additionally adjusted for insurance status and ethnicity, respectively.

To examine HbA_1c_ change within the Pilot-4T cohort, Figure 2 presents adjusted slopes of HbA_1c_ increase from 4 to 12 months by ethnicity (adjusted for age, sex, and insurance) and insurance status (adjusted for age, sex, and ethnicity). By 12 months, HbA_1c_ increased more for Hispanic youths than non-Hispanic youths (slope, 1.56 [95% CI, 1.36-1.77] vs 1.40 [1.21-1.57]; *P* = .007; Figure 2A). Youths with public insurance had a greater HbA_1c_ increase compared with youths with private insurance (slope, 1.63 [95% CI, 1.43-1.83] vs 1.38 [1.20-1.55]; *P* < .001; Figure 2B).

More youths in the Pilot-4T cohort met HbA_1c_ targets (<7.5% and <7.0%) compared with the historical cohort. As shown in eFigure 2 in Supplement 1, a greater proportion of youths in the Pilot-4T cohort achieved a target of less than 7.0% at 12 months postdiagnosis (Hispanic vs non-Hispanic ethnicity: 47.0% vs 54.0%; public vs private insurance: 47.0% vs 57.0%) compared with historical subgroups (Hispanic vs non-Hispanic ethnicity: 24.0% vs 30.0%; public vs private insurance: 19.0% vs 30.0%). Similarly, youths in the Pilot-4T cohort achieved a target of less than 7.5% at 12 months postdiagnosis more often (Hispanic vs non-Hispanic ethnicity: 47.0% vs 73.0%; public vs private insurance: 53.0% vs 71.0%) compared with historical subgroups (Hispanic vs non-Hispanic ethnicity: 35.0% vs 49.0%; public vs private insurance: 25.0% and 50.0%).

### CGM Metrics by Ethnicity and Insurance in the Pilot-4T Cohort

**Figure 3.**
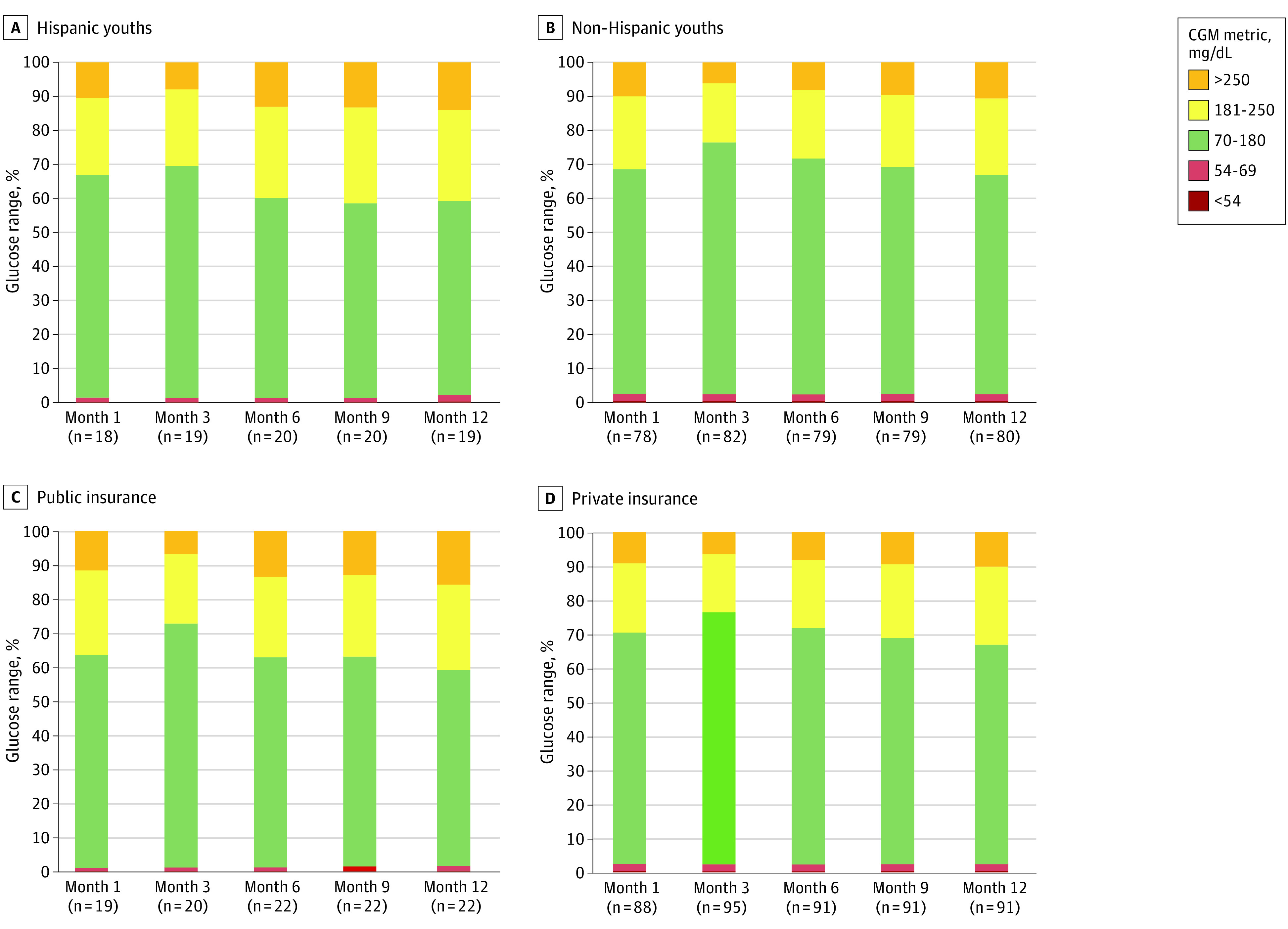
Continuous Glucose Monitoring (CGM) Metrics for Youths in the Pilot-4T Cohort Stratified by Ethnicity and Insurance Status A and B, Hispanic (A) or non-Hispanic (B) ethnicity over the 12-month study period. C and D, Public insurance (C) or private insurance (D) status over the 12-month study period. The CGM metrics included sensor glucose time in range (70-180 mg/dL), hypoglycemia (54-69 mg/dL), and clinically significant hypoglycemia (<54 mg/dL). To convert glucose to mmol/L, multiply by 0.0555.

Data on CGM by ethnicity and insurance over the 12-month study period mirrored HbA_1c_ trajectories (Figure 3). The TIR improved for all youths in the Pilot-4T cohort until the 3-month mark, after which the TIR declined throughout the remainder of the study period. At 12 months, the TIR was 57.0% for Hispanic youths and those with public insurance and 65.0% for non-Hispanic youths and those with private insurance. When evaluating time below range, LOESS figures showed descriptive differences in the percentage of time between 54 and 69 mg/dL by ethnicity (Hispanic vs non-Hispanic: 1.5% vs 2.0%) and by insurance (public vs private: 1.0% vs 2.0%) (eFigure 3 in Supplement 1). However, descriptive differences in the percentage of time spent in clinically significant hypoglycemia (<54 mg/dL) were not observed by ethnicity (Hispanic vs non-Hispanic: 0.3% vs 0.5%) or by insurance (public vs private: 0.3% vs 0.5%) (eFigure 4 in Supplement 1).

## Discussion

The results of this prospective, interventional, pragmatic cohort study suggest that expanding CGM access similarly improved HbA_1c_ by both ethnicity and insurance status but did not eliminate HbA_1c_ disparities among youths with T1D. Inequitable access in diabetes technology has widened disparities in the last decade for underrepresented youths.^3,4,16^ Unlike prior registry findings that show worsening HbA_1c_ disparity,^3^ this study reports a strategy to equitably incorporate CGM for youths, resulting in improvements in HbA_1c_ for all participants. Of equal importance is that access to CGM alone did not eliminate the HbA_1c_ disparity by ethnicity or insurance entirely. These data suggest that access to and use of insulin pumps may together play a role in bridging the HbA_1c_ gap, particularly among Hispanic youths. These findings support the expansion of coverage for early and sustained access to CGM for youths with T1D. Payer coverage, social determinants of health, medical racism, and health policy are likely contributors to the persistent inequities observed in the Pilot-4T cohort.^5,14,35^ These data add to the accumulating evidence base that identifies equitable access to diabetes technology as a modifiable risk factor in T1D and a strategy to bridge diabetes disparities.^3,4,14,36^ In addition to ensuring equitable CGM access, underrepresented youths would benefit from addressing social determinants of health, medical and structural racism, and health policy, as these are likely contributors to the persistent inequities observed among youths with T1D.^14,35^

Although we demonstrated similar improvements in HbA_1c_ for all youths, this intervention did not close the HbA_1c_ gap by ethnicity or insurance completely. In this pilot study, protocols may not have been adequately optimized for remote monitoring (dose recommendations after CGM data review delivered via MyChart), and included issues with internet connectivity, device access, English proficiency, literacy, and numeracy, which may have played a role in this disparity. Remote monitoring utilized medical record messaging for the clinical care team to communicate with the patient in between routine diabetes clinic visits and conferred a 0.15% improvement in HbA_1c_ among the Pilot-4T cohort.^22,37^ Families who used only Dexcom receivers would have had limitations to passive data upload. Emerging studies report that medical record messaging is less utilized and accepted by historically underrepresented individuals, including those from racial and ethnic minority, low-income, and limited English proficiency groups.^38,39,40^ The lack of linguistically and culturally congruent clinicians, a well-documented national problem,^41,42,43^ may also have played a role. Finally, providing access to CGM, while an important step, does not overcome the structural and social determinants of health, medical racism, health policy, and language barriers that impact health outcomes.^14,35^ Achieving equity in T1D health outcomes will require a multifaceted approach with equitable access to CGM for all as a first step.^4,14,35,36^

A key strength of our study design was to approach all patients who were eligible to participate. We intentionally removed clinician discretion in CGM initiation or study enrollment to mitigate the role of clinician implicit bias.^9,13^ Clinician implicit bias plays a role in the provision of diabetes technology.^9,13,14,35,41^ Studies report that clinician bias against public insurance increases with years of practice, and race and ethnicity–mediated bias paradoxically increases with clinician confidence to recognize one’s own bias.^9,13^ Consistent with guidelines,^29,44^ we standardized and structured education to both start and follow-up CGM by certified diabetes care and education specialists, as standardization is considered a strategy to combat implicit bias.^41^

Youths with public insurance have greater barriers to the approval and continued use of CGM^5,6^ and have an increase in HbA_1c_ in association with interruptions to CGM use.^5^ In the US, public insurance policies are typically more stringent for CGM coverage than those of private payers.^5,6^ With grant funding and philanthropic support, the Pilot-4T study bridged gaps in CGM use either due to faulty CGM sensors, delay in insurance-related approval of CGM, or any delay in shipment of CGM supplies. These procedures allowed for the minimization, but not elimination, of system-level drivers of interruptions in CGM use. Challenges with troubleshooting faulty CGM supplies, a barrier to sustained CGM use that disproportionality burdens Hispanic youths and youths with public insurance,^45,46,47^ likely influenced CGM utilization. We hypothesize that the lower percentage of CGM wear-time observed among Hispanic youths and youths with public insurance in the Pilot-4T cohort is an artifact of CGM receivers used in these groups. Although CGM use appeared lower for Hispanic youths and youths with public insurance, a deeper investigation into the data consistent with principles of research equity^48^ suggested that differences in CGM wear-time were mediated by receiver use, not by ethnicity or insurance. Receivers of CGM do not have cloud connectivity and require manual upload of data, which could lead to underreporting of wear-time.

As previously demonstrated,^49^ hypoglycemia rates were low in this cohort. All subgroups achieved hypoglycemia targets (<4.0% time spent <70 mg/dL and <1.0% time spent <54 mg/dL), suggesting that aiming for increased TIR is not inconsistent with achieving hypoglycemia target recommendations.^50^ Hispanic youths and youths with public insurance in the Pilot-4T cohort had lower TIR and less time spent between 54 and 69 mg/dL. These results suggest that Hispanic youths and youths with public insurance in our cohort may benefit from addressing concerns around hypoglycemia to better achieve increased TIR. Study and clinical staff should set similar hypoglycemia goals for all youths. Culturally congruent communication and goal setting by clinicians, youths, and family members may be an important component in bridging HbA_1c_ disparities in addition to providing equitable CGM access.

### Limitations

This study should be interpreted in the context of several limitations, including the exploratory nature of the analyses and the single-site study design. Given that the Pilot-4T study was powered to address the overall efficacy of the 4T program relative to standard of care, we were interested in exploring the interaction between ethnicity and insurance. We evaluated differences in HbA_1c_ and CGM metrics by ethnicity alone without evaluating racial differences. We did not have any individuals who identified as non-Hispanic Black in our cohort, which limits generalizability. We were not able to stratify HbA_1c_ changes in participants who were in remote monitoring by ethnicity or insurance status due to limitations in sample size. However, for those individuals who enrolled in remote monitoring, we ensured cloud connectivity and the ability to engage in remote monitoring by providing smart devices if needed. Although we offered smart devices with internet connectivity for families who received remote monitoring, we observed a “digital divide” in their use, which has been well documented in minoritized populations.^51,52^ Despite these limitations, Hispanic youths and youths with public insurance, who are underrepresented in research,^53^ allowed us to explore aspects of care unique to these groups. Given the increasing incidence and prevalence of T1D among Hispanic or Latinx youths, these data are particularly insightful for this growing proportion of new-onset T1D.^54^ These data provide evidence to suggest the association of early and consistent access to CGM with glycemic outcomes for all youths with T1D in the US, similar to a national program in Australia.^36^

## Conclusions

The findings of this cohort study suggest that universal access to CGM at diabetes diagnosis was associated with an improvement in HbA_1c_ for all participants independent of ethnicity and insurance status but not with elimination of disparities in our Pilot-4T cohort. This finding is in contrast with prior data demonstrating that historically underrepresented youths are often left behind during innovations, resulting in poorer health outcomes.^3,55^ Payers, clinicians, and technology developers should strive to address these gaps in diabetes technology access and to better identify and address drivers of disparities in HbA_1c_ outcomes.

## Notes

Supplement 1. **eTable.** Standardized Mean Difference With 95% CIs by Ethnicity and Insurance **eFigure 1.** Wear-Time Stratified by CGM Download Device Evaluated by Ethnicity and Insurance Status **eFigure 2.** Proportion of Youth in Historical and 4T Cohorts, Stratified by Ethnicity and Insurance Status, Achieving Target HbA_1c_ **eFigure 3.** Percent Time Spent Between 54 and 69 mg/dL, Stratified by Ethnicity and Insurance Status **eFigure 4.** Percent Time in Severe Hypoglycemia (<54 mg/dL), Stratified by Ethnicity and Insurance Status

Supplement 2.
Nonauthor Collaborators

Supplement 3.
Data Sharing Statement
