# Supplementary material for: Disparities in Hemoglobin A1c Levels in the First Year After Diagnosis Among Youths With Type 1 Diabetes Offered Continuous Glucose Monitoring
Source: JAMA Netw Open. 2023 Apr 19;6(4):e238881. doi: 10.1001/jamanetworkopen.2023.8881 (PMC10116368; doi:10.1001/jamanetworkopen.2023.8881)
Supplement: Supplement 1. — eTable. Standardized Mean Difference With 95% CIs by Ethnicity and Insurance eFigure 1. Wear-Time Stratified by CGM Download Device Evaluated by Ethnicity and Insurance Status eFigure 2. Proportion of Youth in Historical and 4T Cohorts, Stratified by Ethnicity and Insurance Status, Achieving Target HbA1c eFigure 3. Percent Time Spent Between 54 and 69 mg/dL, Stratified by Ethnicity and Insurance Status eFigure 4. Percent Time in Severe Hypoglycemia (<54 mg/dL), Stratified by Ethnicity and Insurance Status [file jamanetwopen-e238881-s001.pdf]

Supplementary Online Content

Addala A, Ding V, Zaharieva DP, et al. Disparities in hemoglobin A_{1c} levels in the first year after diagnosis among youths with type 1 diabetes offered continuous glucose monitoring. *JAMA Netw Open*. 2023;6(4):e238881. doi:10.1001/jamanetworkopen.2023.8881

eTable. Standardized Mean Difference With 95% CIs by Ethnicity and Insurance

eFigure 1. Wear-Time Stratified by CGM Download Device Evaluated by Ethnicity and Insurance Status

eFigure 2. Proportion of Youth in Historical and 4T Cohorts, Stratified by Ethnicity and Insurance Status, Achieving Target HbA_{1c}

eFigure 3. Percent Time Spent Between 54 and 69 mg/dL, Stratified by Ethnicity and Insurance Status

eFigure 4. Percent Time in Severe Hypoglycemia (<54 mg/dL), Stratified by Ethnicity and Insurance Status

This supplementary material has been provided by the authors to give readers additional information about their work.

© 2023 Addala A et al. *JAMA Network Open*.

eTable. Standardized Mean Difference With 95% CIs by Ethnicity and Insurance

	**Ethnicity (Hispanic minus non-Hispanic)**	**Insurance (Public minus Private)**
**Baseline characteristic**	SMD [95% CI]	SMD [95% CI]
Age at onset	0.52 [0.10, 0.95]	0.46 [0.05, 0.86]
Sex	0.53 [0.11, 1.0]	0.37 [-0.04, 0.77]
Race	1.3 [0.85, 1.7]	0.45 [0.04, 0.85]
HbA1c (%) at diagnosis	-0.19 [-0.62, 0.23]	-0.01 [-0.41, 0.39]
Insurance type	1.1 [0.64, 1.5]	N/A
Primary language	0.89 [0.46, 1.3]	0.54 [0.13, 0.95]

© 2023 Addala A et al. *JAMA Network Open*.

eFigure 1. Wear-Time Stratified by CGM Download Device Evaluated by Ethnicity and Insurance Status

A

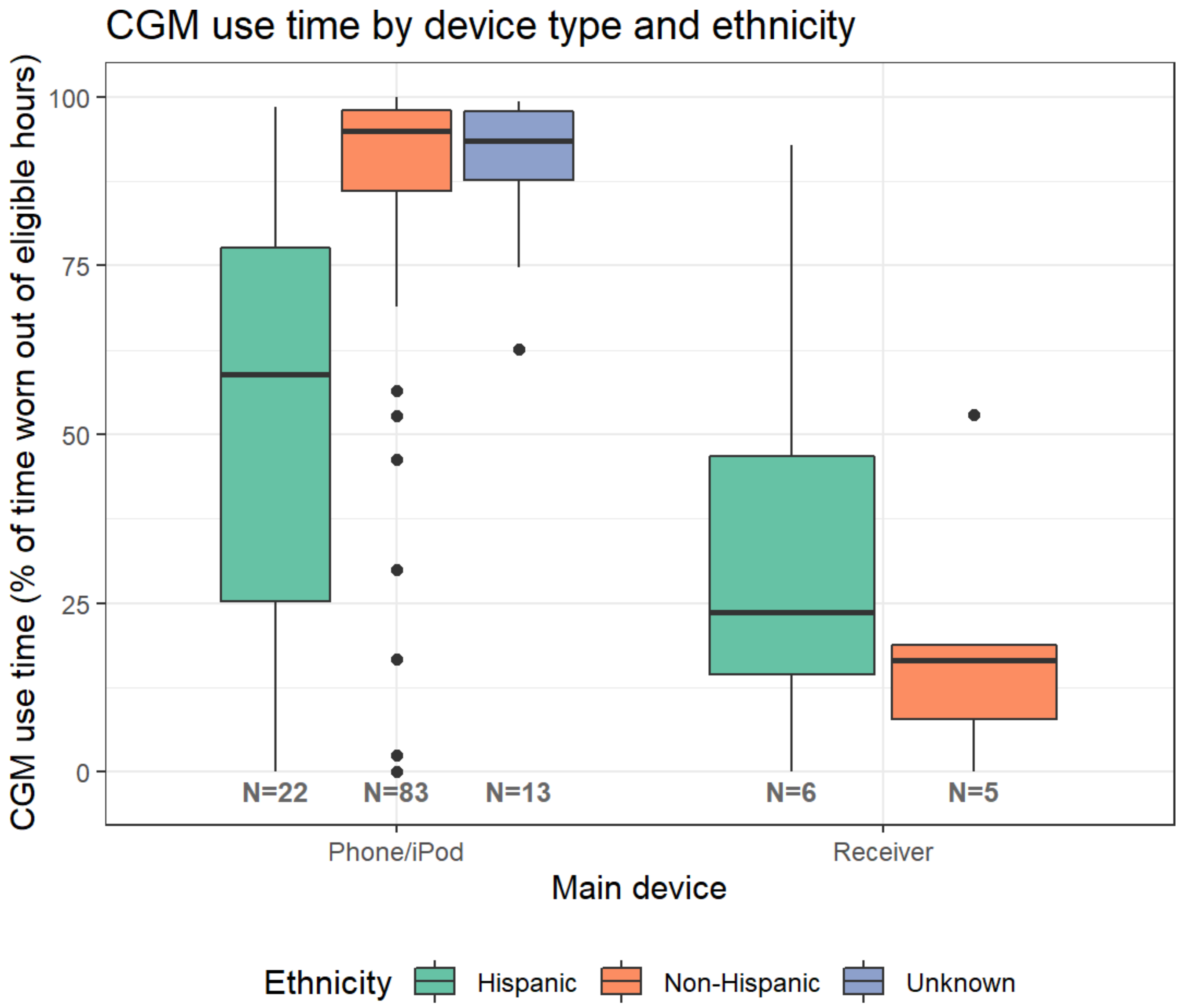

Note: Dexcom G6 data through 2021-07-07

© 2023 Addala A et al. *JAMA Network Open*.

B

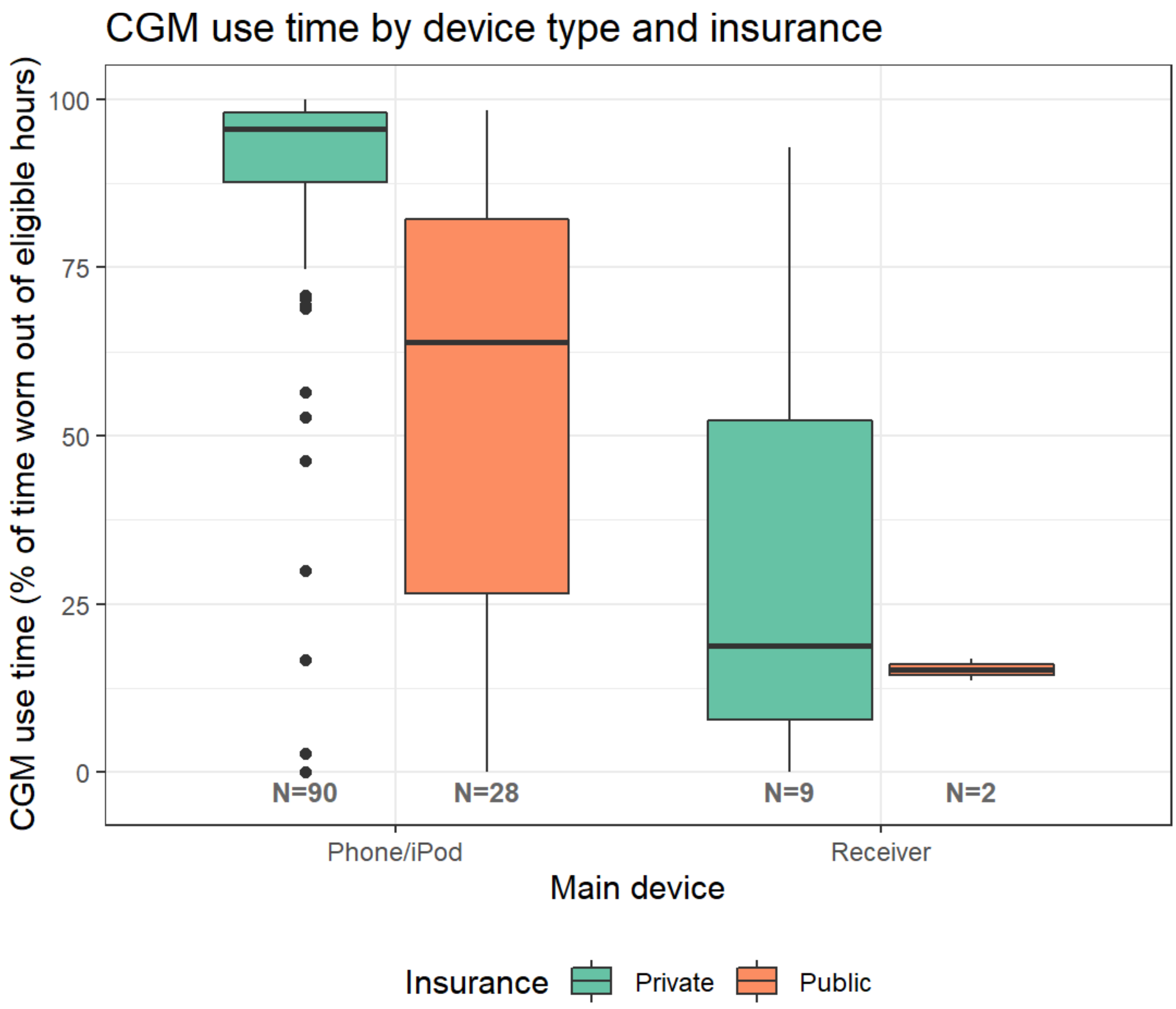

Note: Dexcom G6 data through 2021-07-07

© 2023 Addala A et al. *JAMA Network Open*.

eFigure 2. Proportion of Youth in Historical and 4T Cohorts, Stratified by Ethnicity and Insurance Status, Achieving Target HbA_{1c}

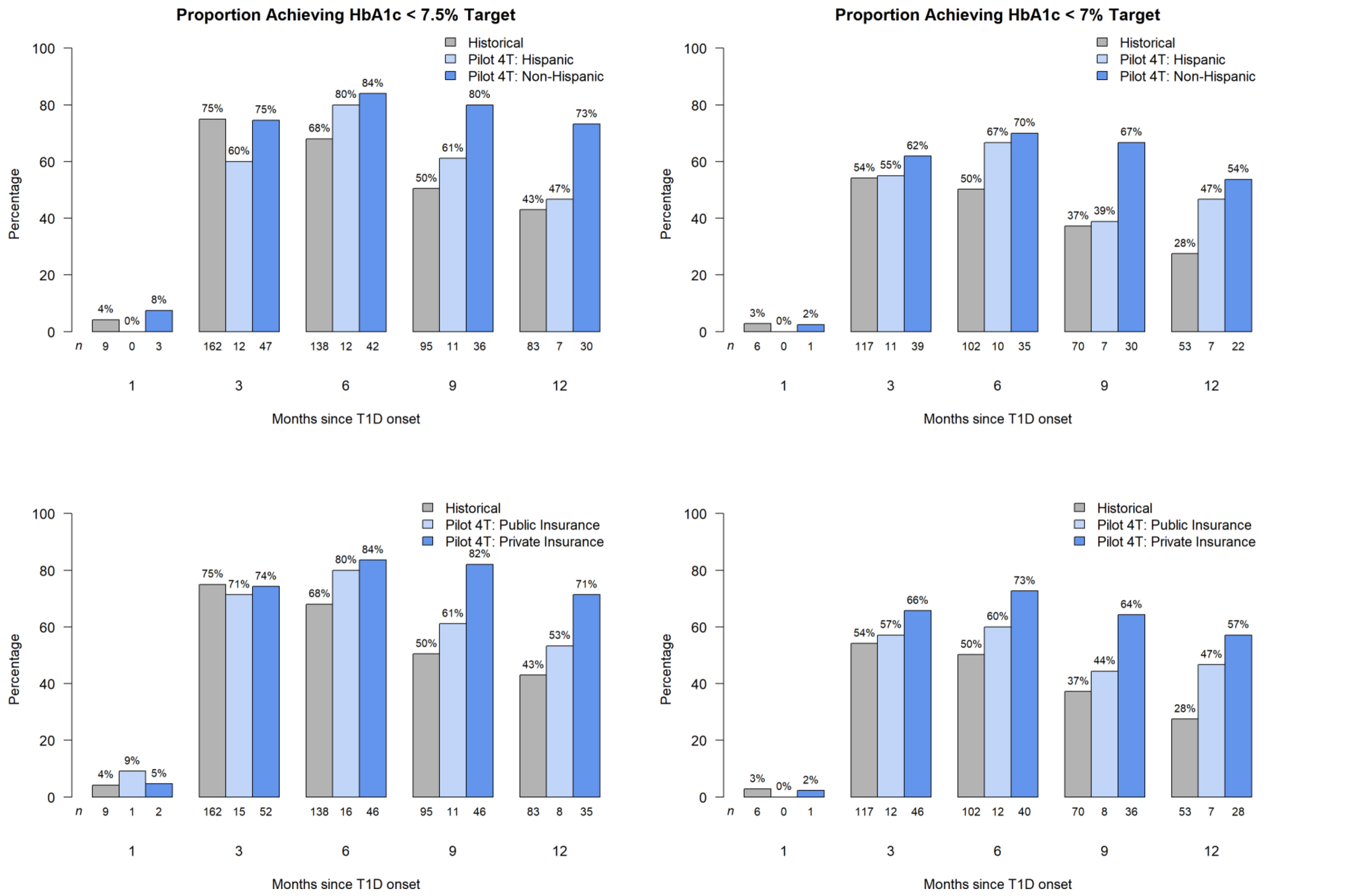

Legend: Proportion of youth in the historical and 4T cohorts meeting HbA1c targets of 7.5% (the HbA1c targets at the time of the start of study) and 7.0% (the current HbA1c targets). Panels A and B compare the proportion of youth in the historical and 4T cohort, stratified by ethnicity, meeting the target of 7.5% and 7.0% respectively. Panels C and D compare the proportion of youth in the historical and 4T cohort, stratified by insurance status, meeting the target of 7.5% and 7.0% respectively.

© 2023 Addala A et al. *JAMA Network Open*.

eFigure 3. Percent Time Spent Between 54 and 69 mg/dL, Stratified by Ethnicity and Insurance Status

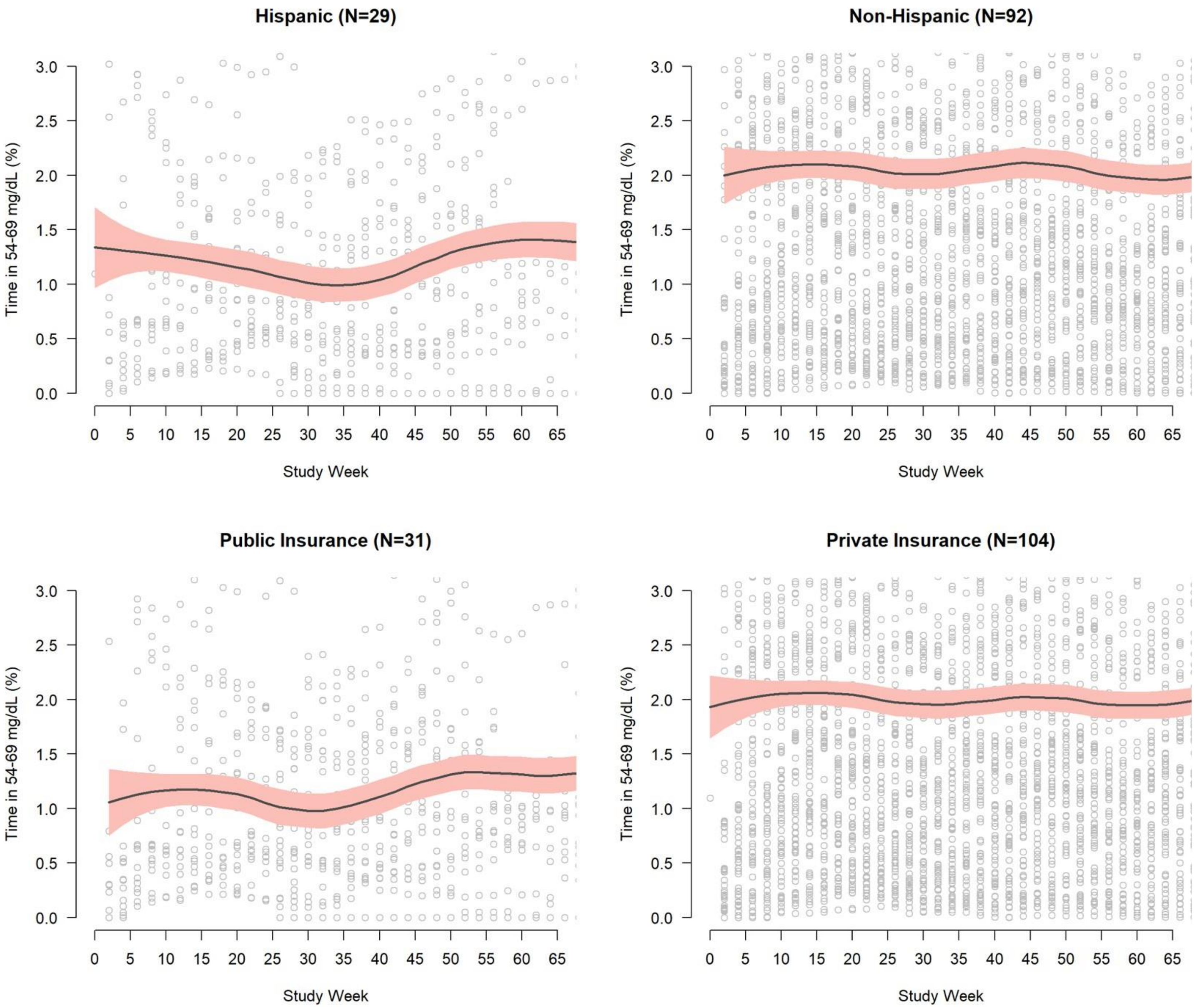

Legend: Percent time spent between 54-69mg/dL in the 4T cohort by ethnicity (panels A and B) and insurance status (panels C and D) over the 12-month study period.

© 2023 Addala A et al. *JAMA Network Open*.

eFigure 4. Percent Time in Severe Hypoglycemia (<54 mg/dL), Stratified by Ethnicity and Insurance Status

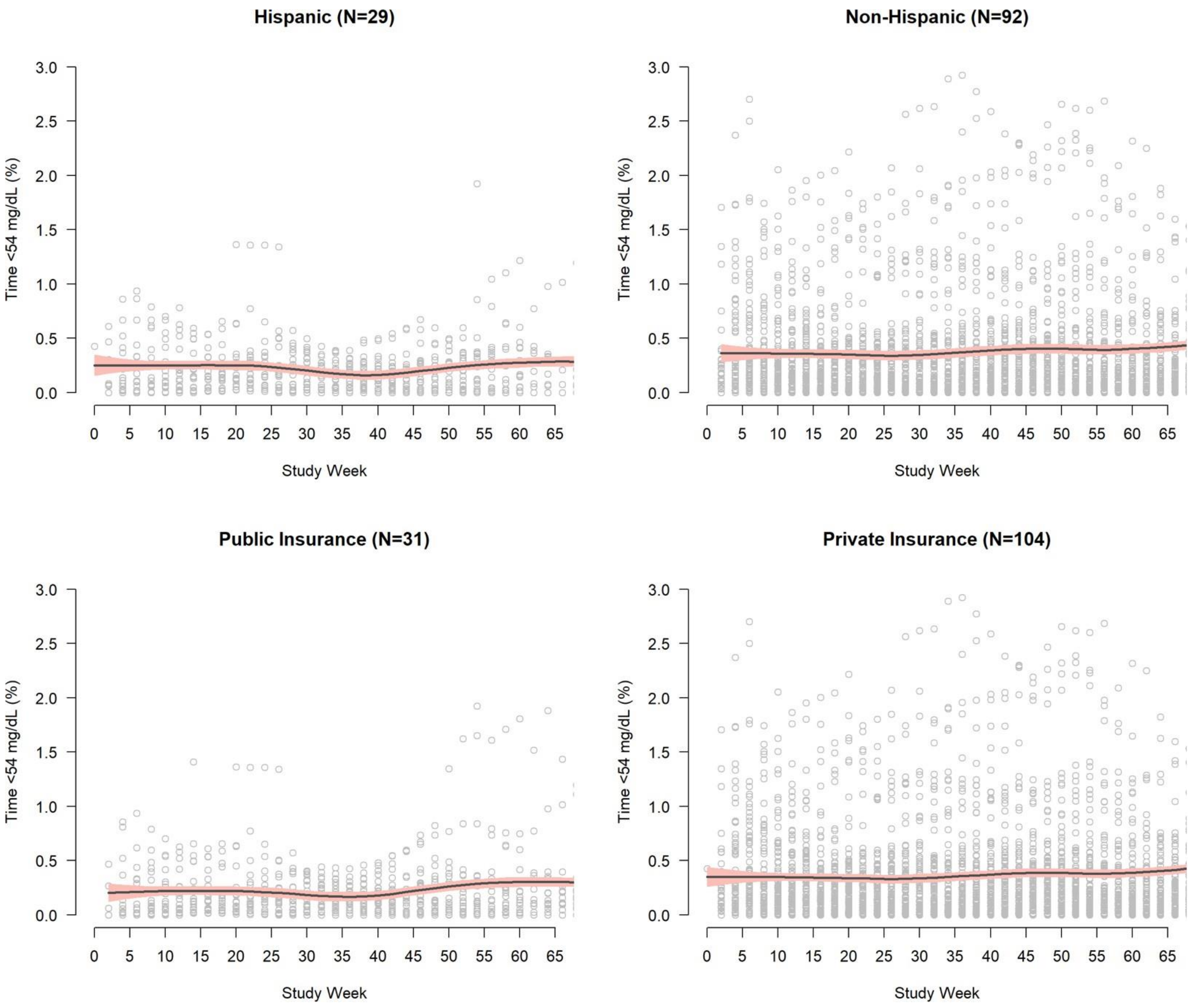

Legend: Percent time spent <54mg/dL in the 4T cohort by ethnicity (panels A and B) and insurance status (panels C and D) over the 12-month study period.

© 2023 Addala A et al. *JAMA Network Open*.
