# Supplementary material for: Disparities in Hemoglobin A1c Levels in the First Year After Diagnosis Among Youths With Type 1 Diabetes Offered Continuous Glucose Monitoring
Source: JAMA Netw Open. 2023 Apr 19;6(4):e238881. doi: 10.1001/jamanetworkopen.2023.8881 (PMC10116368; doi:10.1001/jamanetworkopen.2023.8881)
Supplement: Supplement 2. — Nonauthor Collaborators [file jamanetwopen-e238881-s002.pdf]

Supplemental Online Content: Nonauthor Collaborators
*First name, last name, and suffix (if applicable) are required and will appear in PubMed.

***Group Name(s): Teamwork, Targets, Technology, and Tight Control (4T) Study Group**							
***First Name and Middle Initial(s)**	***Last Name**	***Suffix (eg, Jr, III)**	Academic Degrees	Institution	Location (city, state/province, country)	Role or Contribution, eg, chair, principal investigator	Group (if more than 1 Group listed in the byline) and/or Subgroup (eg, Steering Committee)
Nora	Arrizon-Ruiz			Stanford University	Stanford, CA, USA	study coordinator/data collection	
Erica	Pang		BS	Stanford University	Stanford, CA, USA	study coordinator/data collection	
Ana	Cortes		BS	Stanford University	Stanford, CA, USA	study coordinator/data collection	
Andrea	Bonilla-Ospina		BA	Stanford University	Stanford, CA, USA	study coordinator/data collection	
Rachel	Tam		BA	Stanford University	Stanford, CA, USA	study coordinator/data collection	
Ilenia	Balistreri		BS	Stanford University	Stanford, CA, USA	study coordinator/data collection	
Alondra	Loyola		BS	Stanford University	Stanford, CA, USA	study coordinator/data collection	
Noor	Alramahi		BA	Stanford University	Stanford, CA, USA	study coordinator/data collection	
Eliana	Frank		BS	Stanford University	Stanford, CA, USA	clinical research manager	
Jeannine	Leverenz		RN, CDCES	Stanford University	Stanford, CA, USA	clinical team	
Piper	Sagan		RN, CDCES	Stanford University	Stanford, CA, USA	clinical team	
Anjoli	Martinex-Singh		RD, CDCES	Stanford University	Stanford, CA, USA	clinical team	
Annette	Chmielewski		RD, CDCES	Stanford University	Stanford, CA, USA	clinical team	
Barry	Conrad		RD, CDCES	Stanford University	Stanford, CA, USA	clinical team	
Kim	Clash		NP	Stanford University	Stanford, CA, USA	clinical team	
Julie	Senaldi		RN, CDCES	Stanford University	Stanford, CA, USA	clinical team	
Molly	Tanenbaum		PhD	Stanford University	Stanford, CA, USA	clinical team	
Ransalu	Senanayake		PhD	Stanford University	Stanford, CA, USA	SURF research team	
Ryan	Pei		MS	Stanford University	Stanford, CA, USA	SURF research team	
Esli	Osmanlliu		MD	Stanford University	Stanford, CA, USA	SURF research team	
Annie	Chang		BS	Stanford University	Stanford, CA, USA	SURF research team	
Paul	Dupenloup		MS	Stanford University	Stanford, CA, USA	SURF research team	
Jamie	Kurtzig		BS	Stanford University	Stanford, CA, USA	SURF research team	
Victor	Ritter		PhD	Stanford University	Stanford, CA, USA	Statisical team	
Blake	Shaw		MS	Stanford University	Stanford, CA, USA	Statisical team	
Emily	Fox		PhD	Stanford University	Stanford, CA, USA	Computational Science research team	
Carlos	Guestrin		PhD	Stanford University	Stanford, CA, USA	Computational Science research team	
Johannes	Ferstad		PhD	Stanford University	Stanford, CA, USA	Computational Science research team	
Juan	Langlios		MS	Stanford University	Stanford, CA, USA	Computational Science research team	

Supplemental Online Content: Nonauthor Collaborators
*First name, last name, and suffix (if applicable) are required and will appear in PubMed.

***First Name and Middle Initial(s)**	***Last Name**	***Suffix (eg, Jr, III)**	Academic Degrees	Institution	Location (city, state/province, country)	Role or Contribution, eg, chair, principal investigator	Group (if more than 1 Group listed in the byline) and/or Subgroup (eg, Steering Committee)
Alex	Wang		MS	Stanford University	Stanford, CA, USA	Computational Science research team	
Josesph	Futoma		PhD	Stanford University	Stanford, CA, USA	Computational Science research team	
Matthew	Levine		PhD	Stanford University	Stanford, CA, USA	Computational Science research team	
Arpita	Singhal		BS	Stanford University	Stanford, CA, USA	Computational Science research team	
Yujin	Jeong		BS	Stanford University	Stanford, CA, USA	Computational Science research team	
Matthew	McKay		MS	Stanford University	Stanford, CA, USA	Computational Science research team	
Nataglie	Pageler		MD	Stanford University	Stanford, CA, USA	Informatics research team	
Simrat	Ghuman		PhD	Stanford University	Stanford, CA, USA	Informatics research team	
Michelle	Wiedmann		BS	Stanford University	Stanford, CA, USA	Informatics research team	
Connor	Brown		BS	Stanford University	Stanford, CA, USA	Informatics research team	
Bredan	Watkins		MBA	Stanford University	Stanford, CA, USA	Informatics research team	
Glenn	Loving		MS	Stanford University	Stanford, CA, USA	Informatics research team	
Diana	Naranjo		PhD	Stanford University	Stanford, CA, USA	Psychologist	
