# Supplementary material for: Disparities in Hemoglobin A1c Levels in the First Year After Diagnosis Among Youths With Type 1 Diabetes Offered Continuous Glucose Monitoring
Source: JAMA Netw Open. 2023 Apr 19;6(4):e238881. doi: 10.1001/jamanetworkopen.2023.8881 (PMC10116368; doi:10.1001/jamanetworkopen.2023.8881)
Supplement: Supplement 3. — Data Sharing Statement [file jamanetwopen-e238881-s003.pdf]

Data Sharing Statement

Addala. Disparities in Hemoglobin A_{1c} Levels in the First Year After Diagnosis Among Youths With Type 1 Diabetes Offered Continuous Glucose Monitoring. *JAMA Netw Open*. Published April 19, 2023. doi:10.1001/jamanetworkopen.2023.8881

Data
Data available: No

Additional Information
Explanation for why data not available: The datasets include information that are PHI as it currently sits can lead to the identification of the potential participants. Thus the current IRB coverage for this study does not allow data sharing. However, the authors are willing to share non-priviledge data on a case by case bases as appropriate/indicated.
